# Cardiac magnetic resonance outperforms echocardiography to predict subsequent implantable cardioverter defibrillator therapies in ST-segment elevation myocardial infarction patients

**DOI:** 10.3389/fcvm.2023.991307

**Published:** 2023-02-03

**Authors:** Víctor Marcos-Garcés, Nerea Perez, Jose Gavara, Maria P. Lopez-Lereu, Jose V. Monmeneu, Cesar Rios-Navarro, Elena de Dios, Hector Merenciano-González, Ana Gabaldon-Pérez, Ángel Ferrero-De-Loma-Osorio, Ángel Martínez-Brotons, Lourdes Bondanza, Juan Miguel Sánchez-Gómez, Cristina Albiach, Julio Nunez, Antoni Bayés-Genís, Francisco J. Chorro, Ricardo Ruiz-Granell, Vicente Bodi

**Affiliations:** ^1^Department of Cardiology, Hospital Clínico Universitario de Valencia, Valencia, Spain; ^2^INCLIVA Health Research Institute, Valencia, Spain; ^3^Center for Biomaterials and Tissue Engineering, Universitat Politècnica de València, Valencia, Spain; ^4^Cardiovascular Magnetic Resonance Unit, ASCIRES Biomedical Group, Valencia, Spain; ^5^Faculty of Medicine and Odontology, University of Valencia, Valencia, Spain; ^6^Centro de Investigación Biomédica en Red de Enfermedades Cardiovasculares (CIBERCV), Madrid, Spain; ^7^Cardiology Department and Heart Failure Unit, Hospital Universitari Germans Trias i Pujol, Badalona, Spain; ^8^Department of Medicine, Universitat Autònoma de Barcelona, Barcelona, Spain

**Keywords:** myocardial infarction, implantable cardioverter-defibrillator, cardiac magnetic resonance, ventricular tachyarrhythmias, left ventricular ejection fraction

## Abstract

**Background:**

Implantable cardioverter defibrillators (ICD) are effective as a primary prevention measure of ventricular tachyarrhythmias in patients with ST-segment elevation myocardial infarction (STEMI) and depressed left ventricular ejection fraction (LVEF). The implications of using cardiac magnetic resonance (CMR) instead of echocardiography (Echo) to assess LVEF prior to the indication of ICD in this setting are unknown.

**Materials and methods:**

We evaluated 52 STEMI patients (56.6 ± 11 years, 88.5% male) treated with ICD in primary prevention who underwent echocardiography and CMR prior to ICD implantation. ICD implantation was indicated based on the presence of heart failure and depressed LVEF (≤ 35%) by echocardiography, CMR, or both. Prediction of ICD therapies (ICD-T) during follow-up by echocardiography and CMR before ICD implantation was assessed.

**Results:**

Compared to echocardiography, LVEF was lower by cardiac CMR (30.2 ± 9% vs. 37.4 ± 7.6%, *p* < 0.001). LVEF ≤ 35% was detected in 24 patients (46.2%) by Echo and in 42 (80.7%) by CMR. During a mean follow-up of 6.1 ± 4.2 years, 10 patients received appropriate ICD-T (3.16 ICD-T per 100 person-years): 5 direct shocks to treat very fast ventricular tachycardia or ventricular fibrillation, 3 effective antitachycardia pacing (ATP) for treatment of ventricular tachycardia, and 2 ineffective ATP followed by shock to treat ventricular tachycardia. Echo-LVEF ≤ 35% correctly predicted ICD-T in 4/10 (40%) patients and CMR-LVEF ≤ 35% in 10/10 (100%) patients. CMR-LVEF improved on Echo-LVEF for predicting ICD-T (area under the curve: 0.76 vs. 0.48, *p* = 0.04).

**Conclusion:**

In STEMI patients treated with ICD, assessment of LVEF by CMR outperforms Echo-LVEF to predict the subsequent use of appropriate ICD therapies.

## 1. Introduction

Revolutionary advances in treatment of patients presenting with ST-segment elevation acute myocardial infarction (STEMI) during recent decades has led to a spectacular improvement in prognosis (1). However, despite optimized medical therapy, and in most cases revascularization, the risk of sudden cardiac death is substantial (2, 3).

Left ventricular ejection fraction (LVEF) is the cornerstone for non-invasive risk stratification after STEMI and the main parameter to select patients to undergo prophylactic implantable cardioverter-defibrillator (ICD) implantation, which decreases the risk of sudden cardiac death by treating life-threatening arrhythmias as they arise (4). Current guidelines recommend ICD implantation to reduce sudden cardiac death in patients with New York Heart Association (NYHA) class II–III and LVEF ≤ 35% despite optimal medical therapy for > 3 months and at least ≥ 6 weeks (≈40 days) after STEMI (4, 5).

Nevertheless, most patients treated with an ICD will never require appropriate ICD therapies (ICD-T). In a cohort including five landmark ICD trials, barely one in five patients (18%) were treated with ICD-T during follow-up (6), and the rate was even lower (2.6% at 30 months) among primary prevention patients in a contemporary registry (7). Although ICD-T could be considered potentially lifesaving in those cases, there is an unmet need for strategies aimed at better selection of patients who really benefit from ICD implantation.

Quantification of the scar zone after STEMI and more precise measurement of LVEF could potentially improve patient selection. LVEF by echocardiography (Echo) is routinely performed both at predischarge and during follow-up in most patients. However, cardiac magnetic resonance (CMR) imaging provides the gold standard measurement of LVEF (8, 9) and major crossover between LVEF categories have been shown when Echo and CMR values are compared (10). Additionally, CMR can characterize infarct size (IS), an important predictor of arrhythmic risk (11). However, the utility of CMR before ICD implantation has neither been established in clinical practice nor studied in specific trials, so its usefulness in this scenario is unknown.

Against this background, we aimed to compare LVEF by Echo and CMR measured before ICD implantation for ability to predict ICD-T in a STEMI population treated with ICD in primary prevention.

## 2. Materials and methods

### 2.1. Study group

This study was derived from an ongoing, single-center prospective registry including all patients discharged for a first reperfused STEMI between 2004 and 2021 who were treated with primary percutaneous coronary intervention (pPCI) and followed in a specific outpatient clinic in our hospital. Clinical management was as recommended in specific STEMI guidelines (5). Patient informed consent was obtained. The study was approved by the local Human Research Ethics Committee and complied with the 1975 Declaration of Helsinki guidelines.

Patient characteristics including Global Registry of Acute Coronary Events (GRACE) and TIMI (Thrombolysis in Myocardial Infarction) scores, Killip class at admission, peak creatine kinase MB mass and TIMI flow grade in the culprit artery (before and after reperfusion) were recorded.

### 2.2. ICD implantation

Selection criteria for this analysis were patients treated with ICD in primary prevention in which pre-implantation Echo and CMR had been performed. After at least 6 weeks of optimized medical therapy, symptomatic (NYHA class II–III) patients with LVEF ≤ 35% by Echo, CMR or both underwent ICD implantation. Median time from STEMI to ICD implantation was 40.93 (15.43–188.86) weeks. The flowchart of patients and reasons for exclusion can be consulted in Supplementary Figure 1.

A single lead was positioned in the right ventricular apex using transvenous access. Five (9.6%) patients with expected need for pacing due to atrioventricular block were fitted with an additional auricular lead, and an additional left ventricular lead through the coronary sinus was implanted as cardiac resynchronization therapy in three (5.8%) patients. The ICD generator was placed in a prepectoral pocket in the left upper chest.

Standard three-zone programming was initially provided, with approximate ranges of 175–210 bpm for slow ventricular tachycardia, 210–250 bpm for fast ventricular tachycardia, and > 250 bpm for ventricular fibrillation. Subsequent changes in programming were not systematically recorded in the registry. Remote patient monitoring was provided for most patients (*n* = 35, 67.3%) and in-person medical visits were scheduled on a yearly basis. If remote monitoring was not available, in-person follow-up visits were scheduled every 6 months.

### 2.3. Echocardiography

All patients underwent echocardiographic examination before ICD implantation. Median time from Echo to ICD implantation was 13 (3.75–24.86) weeks. Local cardiologists carried out studies, quantified parameters and prospectively included the data in the local database.

LVEF (%), left ventricular (LV) end-diastolic volume (ml) and LV end-systolic volume (ml) were assessed using the biplane method of disks (modified Simpson’s rule). Right ventricle function was estimated by tricuspid annular plane systolic excursion (TAPSE, in mm), which was measured in the apical 4-chamber view using the M-mode. Diastolic mitral flow A and E wave velocities (m/s) were recorded. Left atrium diameter (mm) was also registered.

### 2.4. CMR

All patients were examined with a 1.5 T System (Sonata Magnetom, Siemens, Erlangen, Germany) using a standardized protocol (10, 12) before ICD implantation. Median time from CMR to ICD implantation was 10.43 (3.71–27.86) weeks (difference vs. time from Echo to ICD implantation: *p* = 0.51).

Images were acquired by a phased-array body surface coil during breath-holds and were ECG-triggered. Local cardiologists specialized in CMR imaging with > 15 years of experience and accredited by the European Society of Cardiology interpreted the studies. Images were examined using customized software (Syngo, Siemens, Erlangen, Germany).

Cine images were acquired in two-, three-, and four-chamber views, and in short-axis views using a steady-state free precession sequence (repetition time/echo time: 2.8/1.2 ms; flip angle: 58°; matrix: 256 × 300; field of view: 320 × 270 mm; slice thickness: 7 mm). LVEF (%), LV end-diastolic volume index (ml/m^2^), LV end-systolic volume index (ml/m^2^), and LV mass index (g/m^2^) were calculated by manual planimetry of endocardial and epicardial borders in short-axis view cine images.

Late gadolinium enhancement (LGE) imaging was performed 10 min after administering gadolinium-based contrast in the same locations as in the cine images, using a segmented inversion recovery steady-state free precession sequence (repetition time/echo time: 750/1.26 ms; flip angle: 45°; matrix: 256 × 184; field of view: 340 × 235 mm; slice thickness: 7 mm). Inversion time was adjusted to nullify normal myocardium.

Areas showing LGE were visually quantified by manual planimetry. Infarct size (IS) was assessed as the percentage of LV mass showing LGE. Microvascular obstruction (MVO) was defined as the number of segments displaying a lack of contrast uptake in the tissue core showing late gadolinium enhancement; the 17-segment model was applied.

### 2.5. LVEF and IS categorization

Patients were categorized as LVEF ≤ 35% or LVEF > 35% by Echo and CMR following current recommendations for ICD implantation in primary prevention in ischemic cardiomyopathy (4, 5). Occurrence of the clinical endpoint was analyzed in these LVEF categories.

### 2.6. Endpoint and follow-up

The clinical endpoint of this study was occurrence of life-threatening ventricular tachyarrhythmias requiring appropriate ICD-T [antitachycardia pacing (ATP), cardioversion, or both]. ICD therapies were considered appropriate when ATP, cardioversion or both were used to treat ventricular tachycardia or ventricular fibrillation, and inappropriate when ATP, cardioversion or both were used for treatment of heart rhythms other than ventricular tachycardia or ventricular fibrillation, such as fast atrial fibrillation. Events were prospectively adjudicated by clinical cardiologists *via* periodic review of regional electronic health records and remote home-monitoring systems if applicable.

### 2.7. Statistical analysis

The one-sample Kolmogorov–Smirnov Test was used to test normal data distribution. For continuous parametric variables, data are expressed as mean ± standard deviation and analyzed by Student’s *t*-test. Continuous non-parametric variables are shown as median plus interquartile range and compared with Mann–Whitney U test. Qualitative variables are presented as percentage and compared by Chi-square test or Fisher’s exact test.

The association between variables and time to first ICD-T was assessed by multivariable Cox proportional hazard regression models. Variables with *p*-value < 0.1 in univariate analysis were included as cofactors in multivariate analysis. Results are presented as hazard ratio (HR) and 95% confidence interval (CI).

Receiver operating characteristic curves were computed to analyze the sensitivity, specificity, and positive and negative predictive value of Echo- and CMR-derived LVEF (≤ 35% or > 35%) categories to predict subsequent ICD-T. Areas under the curve for continuous Echo- and CMR-derived LVEF were compared by means of Z test.

Statistical significance was considered for two-tailed *p*-values < 0.05. The SPSS statistical package version 21.0 was used.

## 3. Results

### 3.1. Cohort description

In our cohort of 52 STEMI patients treated with ICD in primary prevention, mean age was 56.56 ± 11 years, most were male (*n* = 46, 88.5%) and smoking was the most prevalent cardiovascular risk factor (*n* = 36, 69.2%). Most patients in our cohort were Caucasian/White (*n* = 49, 94.3%), while a minority were Asian (*n* = 1, 1.9%), North African Black (*n* = 1, 1.9%), or Latin American (*n* = 1, 1.9%). TIMI flow grade 3 after pPCI was achieved in 39 (75%) patients. Mean GRACE risk score was 137.5 ± 36.51 points, largely indicating moderate to high risk. Baseline characteristics of the cohort are depicted in Table 1.

**TABLE 1:** Baseline, Echo, and CMR characteristics of the entire cohort and of patients with and without ICD-T.

	All patients (*n* = 52)	ICD-T (*n* = 10)	No ICD-T (*n* = 42)	*P*-value
**Clinical variables**				
Age (years)	56.56 ± 11	53.6 ± 12.55	57.38 ± 10.64	0.33
Male sex (%)	46 (88.5)	9 (90)	37 (88.1)	1
Diabetes mellitus (%)	14 (26.9)	3 (30)	11 (26.2)	1
Hypertension (%)	25 (48.1)	3 (30)	22 (52.4)	0.3
Hypercholesterolemia (%)	25 (48.1)	7 (70)	18 (42.9)	0.17
Smoker (%)	36 (69.2)	7 (70)	29 (69)	1
Heart rate on admission (bpm)	89.1 ± 20.74	102.8 ± 14.34	85.83 ± 20.82	0.019
Systolic pressure (mmHg)	127.33 ± 25.19	128.1 ± 20.7	127.14 ± 26.36	0.92
Killip class (%)				0.69
1	36 (69.2)	6 (60)	30 (71.4)	
2	10 (19.2)	3 (30)	7 (16.7)	
3	2 (3.8)	0 (0)	2 (4.8)	
4	4 (7.7)	1 (10)	3 (7.1)	
Time to reperfusion (hours)	190 (135–432.5)	482 (194–559)	180 (120–420)	0.23
Peak creatine kinase MB mass (ng/ml)	300 (180–489)	482 (194–559)	300 (141.25–427.5)	0.17
Infarct location (%)				0.82
Anterior	44 (84.6)	9 (90)	35 (83.3)	
Inferior	7 (13.5)	1 (10)	6 (14.3)	
Lateral	1 (1.9)	0 (0)	1 (2.4)	
TIMI flow grade before pPCI (%)				0.16
0	32 (61.5)	4 (40)	28 (66.7)	
1	3 (5.8)	1 (10)	2 (4.8)	
2	9 (17.3)	4 (40)	5 (11.9)	
3	8 (15.4)	1 (10)	7 (16.7)	
TIMI flow grade after pPCI (%)				0.61
0	2 (3.8)	0 (0)	2 (4.8)	
1	0 (0)	0 (0)	0 (0)	
2	11 (21.2)	3 (30)	8 (19)	
3	39 (75)	7 (70)	32 (76.2)	
GRACE risk score	137.5 ± 36.51	143.5 ± 42.73	136.07 ± 35.3	0.57
TIMI risk score	3.38 ± 2.48	3.7 ± 1.95	3.31 ± 2.6	0.66
Residual ST-segment elevation (*n* of derivations)	2.76 ± 1.79	2.33 ± 1.41	2.89 ± 1.89	0.42
QRS duration (ms)	97.73 ± 19.07	99.78 ± 12.95	97.28 ± 20.3	0.73
Left bundle branch block (%)	2 (3.8)	1 (10)	1 (2.4)	0.35
**Echo indices before ICD implantation**				
Echo-LVEF (%)	37.42 ± 7.61	37.6 ± 5.72	37.38 ± 8.05	0.94
Echo-LV end-diastolic volume (ml)	146.78 ± 43.55	135.4 ± 50.61	148.61 ± 42.97	0.54
Echo-LV end-systolic volume (ml)	90.36 ± 30.48	77.4 ± 29.81	92.45 ± 30.54	0.31
TAPSE (mm)	20.83 ± 4.14	22 ± 3.61	20.67 ± 4.25	0.61
E wave velocity (m/s)	0.78 ± 0.31	1.21 ± 0.52	0.72 ± 0.22	0.15
A wave velocity (m/s)	0.65 ± 0.21	0.57 ± 0.35	0.66 ± 0.2	0.56
Left atrium diameter (mm)	38.31 ± 4.78	37.13 ± 4.16	38.57 ± 4.91	0.45
**CMR indices before ICD implantation**				
CMR-LVEF (%)	30.19 ± 9	23.7 ± 7.8	31.74 ± 8.64	0.01
CMR-LV end-diastolic volume index (ml/m^2^)	116.12 ± 32.9	123.6 ± 49.77	114.29 ± 27.9	0.58
CMR-LV end-systolic volume index (ml/m^2^)	82.52 ± 30.83	96.2 ± 46.31	79.1 ± 25.28	0.29
LV mass (g/m^2^)	94.17 ± 19.44	96.29 ± 23.32	93.66 ± 18.82	0.75
Infarct size (% of LV mass)	37.61 ± 12.7	46.88 ± 13.24	35.41 ± 11.67	0.009

bpm, beats per min; CMR, cardiovascular magnetic resonance; Echo, echocardiography; GRACE, Global Registry of Acute Coronary Events; ICD-T, implantable cardioverter-defibrillator therapies; IS, infarct size; LV, left ventricular; LVEF, left ventricular ejection fraction; pPCI, primary percutaneous coronary intervention; TAPSE, tricuspid annular plane systolic excursion; TIMI, Thrombolysis in Myocardial Infarction. In patients with atrial fibrillation at the time of echocardiography, E and A wave velocities were not considered for analyses.

### 3.2. Echo and CMR indices

On pre-ICD Echo and CMR, patients displayed extensive infarction, LV dysfunction, and dilated LV volumes (Table 1). Mean Echo-LVEF was 37.42 ± 7.61% compared to mean 30.19 ± 9% CMR-LVEF. Mean IS measured by CMR was 37.61 ± 12.7% of LV mass. The mean absolute difference of LVEF measured by Echo vs. CMR was -7.23 ± 11.51% (*p* < 0.001). In most patients (*n* = 41) LVEF was lower by CMR than Echo; in these cases, the mean absolute difference was -11.81 ± 7.34% (*p* < 0.001). In a minority of patients (*n* = 11) LVEF was higher by CMR than Echo; mean absolute difference in these cases was 9.8 ± 7.4% (*p* = 0.001). No interaction was found between patients undergoing Echo and CMR ≥ 12 or < 12 weeks apart (*p* = 0.3, Supplementary Figure 2).

### 3.3. Predictors of ICD-T

During a mean follow-up of 6.08 ± 4.16 years (316.04 ± 216.12 weeks), 10 patients underwent appropriate ICD-T: 5 direct shocks to treat very fast ventricular tachycardia (*n* = 3) or ventricular fibrillation (*n* = 2), 3 effective ATP for treatment of ventricular tachycardia, and 2 ineffective ATP followed by shock to treat ventricular tachycardia. A total of 6 patients received 17 additional recurrent ICD-T treatments: 8 direct shocks, 1 effective ATP, and 8 ineffective ATP followed by shock. The rate of appropriate ICD-T during follow-up was 3.16 per 100 person-years.

On univariate analysis, patients with ICD-T during follow-up presented with higher heart rate on admission (102.8 ± 14.34 bpm vs. 85.83 ± 20.82 bpm, *p* = 0.019). No significant differences were noted regarding Echo indices before ICD implantation. However, on preimplantation CMR, patients with ICD-T during follow-up had lower CMR-LVEF (23.7 ± 7.8 vs. 31.74 ± 8.64, *p* = 0.01) and more extensive IS (46.88 ± 13.24 vs. 35.41 ± 11.67, *p* = 0.009) than patients without this adverse outcome.

On multivariable analysis (Supplementary Table 1), first ICD-T could be predicted by CMR-LVEF [HR 0.9 (0.83–0.99) per %, *p* = 0.02] and heart rate on admission [HR 1.05 (1–1.1) per beat per min, *p* = 0.03]. The predictive power of IS was marginally significant [HR 1.05 (0.99–1.12) per % of LV mass, *p* = 0.11]. Pre-ICD Echo-LVEF did not appear to accurately predict use of ICD-T in either univariate or multivariable analyses.

### 3.4. ICD-T stratification by LVEF categories

**FIGURE 1:**
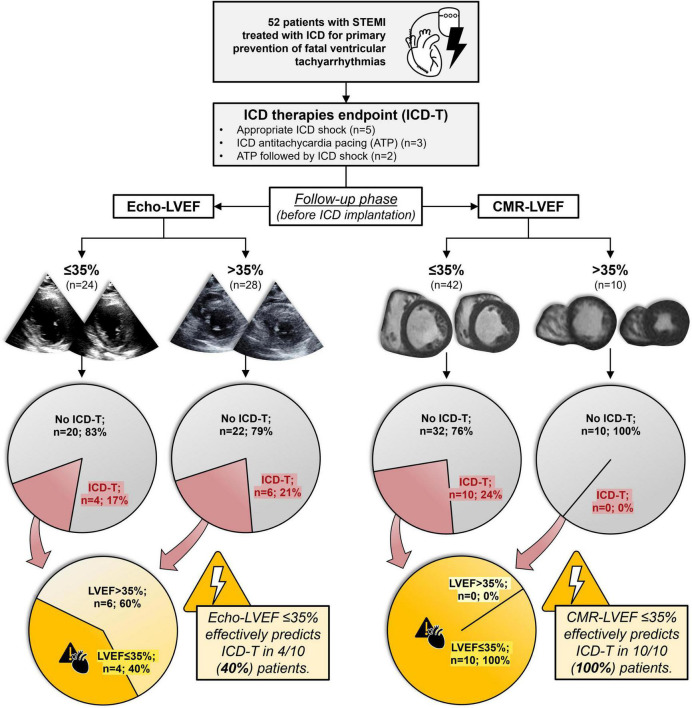
Stratification of ICD-T according to Echo and CMR LVEF categories before ICD implantation. Occurrence of life-threatening ventricular tachyarrhythmias (manifesting as appropriate ICD therapies) was analyzed in a population of STEMI patients treated with ICD for primary prevention. Patients were categorized by pre-ICD LVEF (≤ 35% vs. >35%) *via* Echo or CMR. Compared to Echo-LVEF, CMR-LVEF categories allowed for detection of 100% of ICD-T (when CMR-LVEF was ≤ 35%) and exclusion of ICD-T (when CMR-LVEF was > 35%). ATP, antitachycardia pacing; CMR, cardiac magnetic resonance; Echo, echocardiography; ICD, implantable cardioverter-defibrillator; ICD-T, appropriate implantable cardioverter-defibrillator therapies; LVEF, left ventricular ejection fraction; STEMI, ST-segment elevation acute myocardial infarction.

**FIGURE 2:**
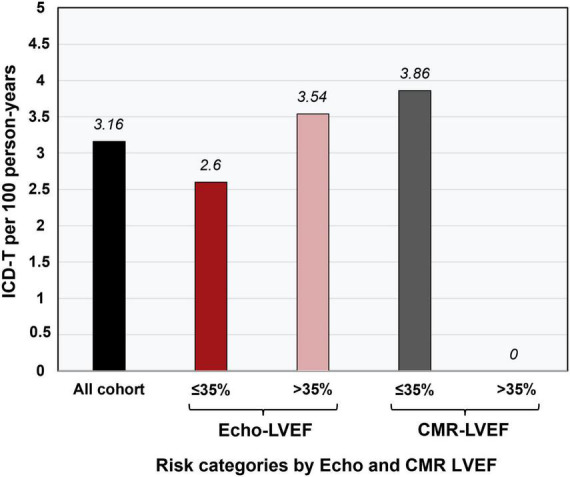
ICD-T per 100 person-years across Echo and CMR LVEF categories before ICD implantation. CMR, cardiac magnetic resonance; Echo, echocardiography; ICD, implantable cardioverter-defibrillator; ICD-T, appropriate implantable cardioverter-defibrillator therapies; LVEF, left ventricular ejection fraction.

Using the recommended cutoff of LVEF ≤ 35% to select patients eligible for ICD implantation in primary prevention, we stratified our cohort into two groups by both pre-ICD Echo and CMR (Figure 1). Using Echo-LVEF, LVEF ≤ 35% identified only 4 out of 10 (40%) patients who received appropriate ICD-T during follow-up. In contrast, CMR-LVEF ≤ 35% before ICD implantation identified 10 out of 10 (100%) patients who underwent appropriate ICD-T during follow-up. In our population of ICD carriers in primary prevention, therefore, Echo-LVEF ≤ 35% had 40% sensitivity, 52.4% specificity, 16.7% positive predictive value and 78.6% negative predictive value for appropriate ICD-T, compared to the 100% sensitivity, 23.8% specificity, 23.8% positive predictive value, and 100% negative predictive value of CMR-LVEF ≤ 35% (Table 2). MACE per 100 person-years across the Echo and CMR LVEF categories is depicted in Figure 2. CMR-LVEF outperformed Echo-LVEF for predicting ICD-T (area under the curve 0.76 vs. 0.48, *p* = 0.04).

**TABLE 2:** ROC curve characteristics of LVEF categories by Echo and CMR before ICD implantation to predict appropriate ICD-T.

Variable	Sensitivity	Specificity	Positive predictive value	Negative predictive value
Echo-LVEF ≤ 35%	40%	52.4%	16.7%	78.6%
CMR-LVEF ≤ 35%	100%	23.8%	23.8%	100%

CMR, cardiovascular magnetic resonance; Echo, echocardiography; ICD-T, implantable cardioverter-defibrillator therapies; IS, infarct size; LVEF, left ventricular ejection fraction; ROC, receiver operating characteristic.

## 4. Discussion

In our population of STEMI patients treated with ICD for primary prevention, the main findings of our study are as follows: (1) LVEF measured by CMR outperformed Echo-LVEF and CMR-IS to predict ICD-T; (2) CMR-LVEF ≤ 35% accurately identified 100% of patients who would require ICD-T (100% sensitivity and negative predictive value), and (3) no ICD-T were registered during follow-up in patients with CMR-LVEF > 35%.

### 4.1. LVEF and ICD in primary prevention

In conjunction with other clinical parameters, LVEF is the cornerstone for early out-of-hospital cardiac arrest prediction in STEMI patients (3). Patients with reduced (< 50%) LVEF have an increased risk of adverse events (10) and sudden cardiac death (3). However, LVEF can fluctuate widely either upward or downward, and together with the magnitude or absence of recovery, LVEF level several weeks after myocardial infarction is a relevant marker of adverse events including sudden cardiac arrest (13, 14). Since a trend toward recovery is usually seen in patients with reduced LVEF, remeasurement of this parameter is fundamental after the acute phase (12, 15). Nonetheless, in clinical practice reassessment rates are relatively low, even in patients with LVEF < 40% in acute phase (16), and this less closely monitored LVEF results in a lower likelihood of being treated with an ICD (17).

### 4.2. Echocardiography vs. CMR for risk stratification

Echocardiography has traditionally been the imaging technique of choice for patient follow-up and specifically for LVEF measurement. Indeed, clinical trials for ICD in primary prevention in ischemic heart disease have mostly relied on echocardiography for measuring LVEF and selecting the most appropriate cut-off for patient selection (18, 19).

Nevertheless, CMR imaging is the current gold standard for accurate and reproducible LVEF measurement (8, 9). Unfortunately, agreement between LVEF values by echocardiography and CMR is poor, especially if patients with LVEF of 35% or less are included (20). This has also been observed specifically in STEMI patients, an effect probably exacerbated by the presence of segmental wall motion abnormalities (10, 21).

### 4.3. LVEF to predict ICD therapies

Basing the decision to implant an ICD in primary prevention solely on LVEF has several limitations as an approach, as well as a relatively low pooled sensitivity (59.1%) and specificity (77.8%) for predicting major arrhythmic events after myocardial infarction (22). In fact, most patients with LVEF ≤ 35% will never require ICD-T if implanted with an ICD. Across five landmark ICD trials, only 18% of patients were treated with ICD-T during follow-up (6). The rate is even lower (2.6% at 30 months) among primary prevention patients in a contemporary registry (7), probably due to improved preventive therapies. The rate of appropriate ICD-T during follow-up was 3.16 per 100 person-years in our cohort, similar to previously published cohorts. As an example, in the Danish ICD register patients with ischemic heart disease and ICD implanted for primary prevention showed an appropriate ICD-T rate of 4.12 per 100 person-years (23).

Furthermore, as most contemporary STEMI patients maintain a relatively preserved LVEF after the acute event, the majority of sudden cardiac deaths and arrhythmic events occur in this subset regardless of their reduced risk of arrhythmic events (24, 25). There is a considerable risk of sudden cardiac arrest in STEMI patients with LVEF 35–50%, a population in which ICD is generally not indicated in primary prevention (26). Indeed, in our study most (60%) appropriate ICD-T occurred in patients with Echo-LVEF > 35%. As previously noted, therefore, Echo-derived LVEF is not a sensitive predictor of ICD-T.

### 4.4. CMR to predict ICD therapies

The efficacy of CMR imaging to predict the use of appropriate therapies in ICD carriers is an understudied area, despite being a non-invasive and safe technique with proven predictive value in STEMI patients (10, 12). Studies have shown that CMR-derived LVEF (27) and IS (27, 28) can predict adverse arrhythmic cardiac events and ICD-T. In fact, CMR-LVEF outperforms Echo-LVEF to predict a composite endpoint of all-cause death or ICD-T in ICD carriers in primary prevention with ischemic and non-ischemic cardiomyopathy (29). Likewise, quantification of myocardial fibrosis and gray zone fibrosis in CMR can predict sudden cardiac death and an arrhythmic endpoint in a mixed (ischemic and non-ischemic) population of cardiac implantable electronic device receivers (30). Interestingly, in patients with no evidence of fibrosis in CMR, sudden cardiac death can be virtually excluded. However, in our STEMI cohort the presence of at least some degree of myocardial necrosis was universal, and CMR-LVEF appears more useful to predict (or exclude) ICD-T during follow-up.

Occurrence of pre-ICD CMR-LVEF ≤ 35% identified all patients (100%) in this cohort who would undergo ICD-T during follow-up, clearly outperforming pre-ICD Echo-LVEF ≤ 35%, which had only 40% sensitivity to detect ICD-T. The presence of CMR-LVEF > 35% thus indicates a low risk of adverse arrhythmic events and can pinpoint a population in which ICD implantation should be withheld. In contrast, the possibility of adverse arrhythmic events could not be ruled out in the subgroup with Echo-LVEF > 35%.

We hypothesize that these findings can be explained by the increased accuracy of LVEF measurement by CMR (i.e., LVEF ≤ 35% by CMR represents a truly reduced LVEF) and the lower LVEF obtained by CMR compared to echocardiography (i.e., LVEF > 35% by CMR represents a truly not-severely reduced LVEF).

In the selection of STEMI patients who could benefit from an ICD in primary prevention, clinicians should weigh up the risk of undertreatment, which would deprive certain patients of potentially life-saving therapy in case of fatal ventricular arrhythmias, against the risk of overtreatment, which would increase provider costs and morbidity associated with complications and inappropriate therapies in patients fitted with a device. Based on our data, a LVEF ≤ 35% cut-off by CMR imaging could accurately identify all patients who would require ICD-T during follow-up, and most importantly, safely exclude ventricular tachyarrhythmias in patients with CMR-LVEF > 35%, in whom theoretically an ICD would render no preventive benefit.

### 4.5. Study limitations

Our cohort is limited in the number of recruited patients and compiled variables. Additionally, patients were included over a long period (between 2004 and 2021) during which time there were variations in acute and chronic STEMI treatment. Only STEMI patients who underwent both echocardiography and CMR before ICD implantation were selected, so the cohort may not be entirely representative of the whole STEMI population. Another drawback is the time difference between Echo and CMR and ICD implantation in our cohort; performing these techniques sequentially over a short period could have allowed better comparison. Patients not undergoing ICD implantation were not studied, which allowed us to accurately analyze the occurrence of ICD-T but limited our results to ICD carriers. Changes in ICD programming were not systematically recorded during follow-up. Furthermore, although most patients showed extensive infarction, underwent CMR study and ischemic etiology could be inferred as the most probable cause, other underlying cardiomyopathies could not be ruled out with absolute certainty. Lastly, the implications of ICD implantation based on CMR-LVEF vs. Echo-LVEF should be explored in specifically designed, prospective, and randomized studies. Due to the observational nature of our study no formal recommendations regarding ICD indication can be inferred.

## 5. Conclusion

In STEMI patients treated with ICD in primary prevention, assessment of LVEF by CMR outperforms Echo-LVEF to predict subsequent appropriate ICD therapies. Occurrence of CMR-LVEF ≤ 35% identified all patients who would undergo ICD therapies (100% sensitivity and negative predictive value). Strategies aimed at selective ICD implantation based on CMR data should be further explored in properly designed studies.

## Data availability statement

The datasets presented in this article are not readily available because of privacy and ethical restrictions. Requests to access the datasets should be directed to VB, vicente.bodi@uv.es.

## Ethics statement

The study was reviewed and approved by the Comité Ético de Investigación con Medicamentos of the Hospital Clínico Universitario de Valencia. The patients/participants provided their written informed consent to participate in this study.

## Author contributions

VM-G, NP, and VB: conceptualization and writing—original draft. VM-G, NP, JG, ML-L, JM, CR-N, ED, HM-G, AG-P, ÁF-D-L-O, ÁM-B, LB, JS-G, and CA: data curation. VM-G, NP, JG, ML-L, JM, CR-N, ED, HM-G, AG-P, ÁF-D-L-O, ÁM-B, LB, JS-G, CA, and VB: formal analysis. JN, AB-G, FC, and VB: funding acquisition. VM-G, NP, JG, ML-L, JM, and VB: investigation and methodology. VM-G, NP, CR-N, and VB: project administration. VM-G, NP, JG, ML-L, JM, CR-N, ED, HM-G, AG-P, ÁF-D-L-O, ÁM-B, LB, JS-G, CA, JN, AB-G, FC, RR-G, and VB: resources. JN, AB-G, FC, RR-G, and VB: supervision and writing—review and editing. VM-G, NP, JG, and VB: validation and visualization. All authors contributed to the article and approved the submitted version.

## References

[1] Nabel E Braunwald E. A tale of coronary artery disease and myocardial infarction. *N Engl J Med.* (2012) 366:54–63. 10.1056/NEJMra1112570 22216842

[2] Fan X Hua W Xu Y Ding L Niu H Chen K Incidence and predictors of sudden cardiac death in patients with reduced left ventricular ejection fraction after myocardial infarction in an era of revascularisation. *Heart.* (2014) 100:1242–9. 10.1136/heartjnl-2013-305144 24895352

[3] Faxén J Jernberg T Hollenberg J Gadler F Herlitz J Szummer K. Incidence and predictors of out-of-hospital cardiac arrest within 90 days after myocardial infarction. *J Am Coll Cardiol.* (2020) 76:2926–36. 10.1016/j.jacc.2020.10.033 33334420

[4] Al-Khatib S Stevenson W Ackerman M Bryant W Callans D Curtis A 2017 AHA/ACC/HRS Guideline for Management of Patients With Ventricular Arrhythmias and the Prevention of Sudden Cardiac Death: A Report of the American College of Cardiology/American Heart Association Task Force on Clinical Practice Guidelines and the Heart Rhythm Society. *Circulation.* (2018) 138:e272–391. 10.1161/CIR.0000000000000614 29084731

[5] Ibanez B James S Agewall S Antunes M Bucciarelli-Ducci C Bueno H 2017 ESC Guidelines for the management of acute myocardial infarction in patients presenting with ST-segment elevationThe Task Force for the management of acute myocardial infarction in patients presenting with ST-segment elevation of the European Society of Cardiology (ESC). *Eur Heart J.* (2018) 39:119–77. 28886621 10.1093/eurheartj/ehx393

[6] Aktaş M Younis A Zareba W Kutyifa V Klein H Daubert J Survival after implantable cardioverter-defibrillator shocks. *J Am Coll Cardiol.* (2021) 77:2453–62. 10.1016/j.jacc.2021.03.329 34016257 PMC8142936

[7] Sabbag A Suleiman M Laish-Farkash A Samania N Kazatsker M Goldenberg I Contemporary rates of appropriate shock therapy in patients who receive implantable device therapy in a real-world setting: From the Israeli ICD Registry. *Heart Rhythm.* (2015) 12:2426–33. 10.1016/j.hrthm.2015.08.020 26277863

[8] Grothues F Smith G Moon J Bellenger N Collins P Klein H Comparison of interstudy reproducibility of cardiovascular magnetic resonance with two-dimensional echocardiography in normal subjects and in patients with heart failure or left ventricular hypertrophy. *Am J Cardiol.* (2002) 90:29–34. 10.1016/S0002-9149(02)02381-0 12088775

[9] Bulluck H Dharmakumar R Arai A Berry C Hausenloy D. Cardiovascular magnetic resonance in acute st-segment-elevation myocardial infarction: Recent advances, controversies, and future directions. *Circulation.* (2018) 137:1949–64. 10.1161/CIRCULATIONAHA.117.030693 29712696 PMC5933067

[10] Marcos-Garces V Gavara J Lopez-Lereu M Monmeneu JV Rios-Navarro C de Dios E Ejection fraction by echocardiography for a selective use of magnetic resonance after infarction. *Circ Cardiovasc Imaging.* (2020) 13:e011491. 10.1161/CIRCIMAGING.120.011491 33297764

[11] Alzuhairi K Lønborg J Ahtarovski K Nepper-Christensen L Kyhl K Lassen J Sub-acute cardiac magnetic resonance to predict irreversible reduction in left ventricular ejection fraction after ST-segment elevation myocardial infarction: A DANAMI-3 sub-study. *Int J Cardiol.* (2020) 301:215–9. 10.1016/j.ijcard.2019.10.034 31748187

[12] Gavara J Marcos-Garces V Lopez-Lereu M Monmeneu J Rios-Navarro C de Dios E Magnetic resonance assessment of left ventricular ejection fraction at any time post-infarction for prediction of subsequent events in a large multicenter STEMI registry. *J Magn Reson Imaging.* (2021) 56:476–87. 10.1002/jmri.27789 34137478

[13] Exner D Kavanagh K Slawnych M Mitchell L Ramadan D Aggarwal S Noninvasive risk assessment early after a myocardial infarction. *J Am Coll Cardiol.* (2007) 50:2275–84. 10.1016/j.jacc.2007.08.042 18068035

[14] Chew D Heikki H Schmidt G Kavanagh K Dommasch M Bloch Thomsen P Change in left ventricular ejection fraction following first myocardial infarction and outcome. *JACC Clin Electrophysiol.* (2018) 4:672–82. 10.1016/j.jacep.2017.12.015 29798797

[15] Søholm H Lønborg J Andersen M Vejlstrup N Engstrøm T Møller J Repeated echocardiography after first ever ST-segment elevation myocardial infarction treated with primary percutaneous coronary intervention – is it necessary? *Eur Heart J Acute Cardiovasc Care.* (2015) 4:528–36. 10.1177/2048872614556000 25318482

[16] Chew D Wilton S Kavanagh K Southern D Tan-Mesiatowsky L Exner DV. Left ventricular ejection fraction reassessment post-myocardial infarction: Current clinical practice and determinants of adverse remodeling. *Am Heart J.* (2018) 198:91–6. 10.1016/j.ahj.2017.11.014 29653653

[17] Pokorney S Miller A Chen A Thomas L Fonarow G de Lemos J Reassessment of cardiac function and implantable cardioverter-defibrillator use among medicare patients with low ejection fraction after myocardial infarction. *Circulation.* (2017) 135:38–47. 10.1161/CIRCULATIONAHA.116.022359 27881561

[18] Moss A Zareba W Hall W Klein H Wilber D Cannom D Prophylactic implantation of a defibrillator in patients with myocardial infarction and reduced ejection fraction. *N Engl J Med.* (2002) 346:877–83. 10.1056/NEJMoa013474 11907286

[19] Bardy G Lee K Mark D Poole J Packer D Boineau R Amiodarone or an implantable cardioverter–defibrillator for congestive heart failure. *N Engl J Med.* (2005) 352:225–37. 10.1056/NEJMoa043399 15659722

[20] Pellikka P She L Holly T Lin G Varadarajan P Pai R Variability in ejection fraction measured by echocardiography, gated single-photon emission computed tomography, and cardiac magnetic resonance in patients with coronary artery disease and left ventricular dysfunction. *JAMA Netw Open.* (2018) 1:e181456. 10.1001/jamanetworkopen.2018.1456 30646130 PMC6324278

[21] Hendriks T Al Ali L Maagdenberg C van Melle J Hummel Y Oudkerk M Agreement of 2D transthoracic echocardiography with cardiovascular magnetic resonance imaging after ST-elevation myocardial infarction. *Eur J Radiol.* (2019) 114:6–13. 10.1016/j.ejrad.2019.02.039 31005178

[22] Bailey J Berson A Handelsman H Hodges M. Utility of current risk stratification tests for predicting major arrhythmic events after myocardial infarction. *J Am Coll Cardiol.* (2001) 38:1902–11. 10.1016/S0735-1097(01)01667-9 11738292

[23] Weeke P Johansen J Jorgensen O Nielsen J Moller M Videbaek R Mortality and appropriate and inappropriate therapy in patients with ischaemic heart disease and implanted cardioverter-defibrillators for primary prevention: data from the Danish ICD Register. *Europace.* (2013) 15:1150–7. 10.1093/europace/eut017 23407630

[24] Dagres N Hindricks G. Risk stratification after myocardial infarction: is left ventricular ejection fraction enough to prevent sudden cardiac death? *Eur Heart J.* (2013) 34:1964–71. 10.1093/eurheartj/eht109 23644180

[25] Bui A Waks J. Risk stratification of sudden cardiac death after acute myocardial infarction. *J Innov Cardiac Rhythm Manage.* (2018) 9:3035–49. 10.19102/icrm.2018.090201 32477797 PMC7252689

[26] Adabag S Zimmerman P Lexcen D Cheng A. Predictors of Sudden Cardiac Arrest Among Patients With Post-Myocardial Infarction Ejection Fraction Greater Than 35%. *J Am Heart Assoc* (2021) 10:e020993. 10.1161/JAHA.121.020993 34259015 PMC8483475

[27] Izquierdo M Ruiz-Granell R Bonanad C Chaustre F Gomez C Ferrero A Value of early cardiovascular magnetic resonance for the prediction of adverse arrhythmic cardiac events after a first noncomplicated ST-segment-elevation myocardial infarction. *Circ Cardiovasc Imaging.* (2013) 6:755–61. 10.1161/CIRCIMAGING.113.000702 23926195

[28] Alexandre J Saloux E Dugué A Lebon A Lemaitre A Roule V Scar extent evaluated by late gadolinium enhancement CMR: a powerful predictor of long term appropriate ICD therapy in patients with coronary artery disease. *J Cardiovasc Magn Reson.* (2013) 15:12. 10.1186/1532-429X-15-12 23331500 PMC3610203

[29] Champ-Rigot L Gay P Seita F Benouda L Morello R Pellissier A Clinical outcomes after primary prevention defibrillator implantation are better predicted when the left ventricular ejection fraction is assessed by cardiovascular magnetic resonance. *J Cardiovasc Magn Reson.* (2020) 22:48. 10.1186/s12968-020-00640-0 32580786 PMC7315498

[30] Leyva F Zegard A Okafor O Foley P Umar F Taylor R Myocardial fibrosis predicts ventricular arrhythmias and sudden death after cardiac electronic device implantation. *J Am Coll Cardiol.* (2022) 79:665–78. 10.1016/j.jacc.2021.11.050 35177196

