# Supplementary material for: Cardiac magnetic resonance outperforms echocardiography to predict subsequent implantable cardioverter defibrillator therapies in ST-segment elevation myocardial infarction patients
Source: Front Cardiovasc Med. 2023 Feb 3;10:991307. doi: 10.3389/fcvm.2023.991307 (PMC9937054; doi:10.3389/fcvm.2023.991307)
Supplement: Supplementary file 1 [file Data_Sheet_1.docx]

Supplementary Material

Cardiac magnetic resonance outperforms echocardiography to predict subsequent implantable cardioverter defibrillator therapies in ST-segment elevation myocardial infarction patients

# Supplementary Tables

**Supplementary Table 1.** Predictors of ICD-T on multivariable analysis.

|  | **Hazard Ratio [95% CI]** | ***p*-value** |
| --- | --- | --- |
| **Heart rate on admission (beats per min)** | 1.05 [1–1.1] | 0.03 |
| **CMR-LVEF (%)** | 0.9 [0.83–0.99] | 0.02 |
| **Infarct size (% of LV mass)** | 1.05 [0.99–1.12] | 0.11 |

Abbreviations. CMR = Cardiovascular magnetic resonance. ICD-T = Implantable cardioverter-defibrillator therapies. LV = Left ventricular. LVEF = Left ventricular ejection fraction.

# Supplementary Figures


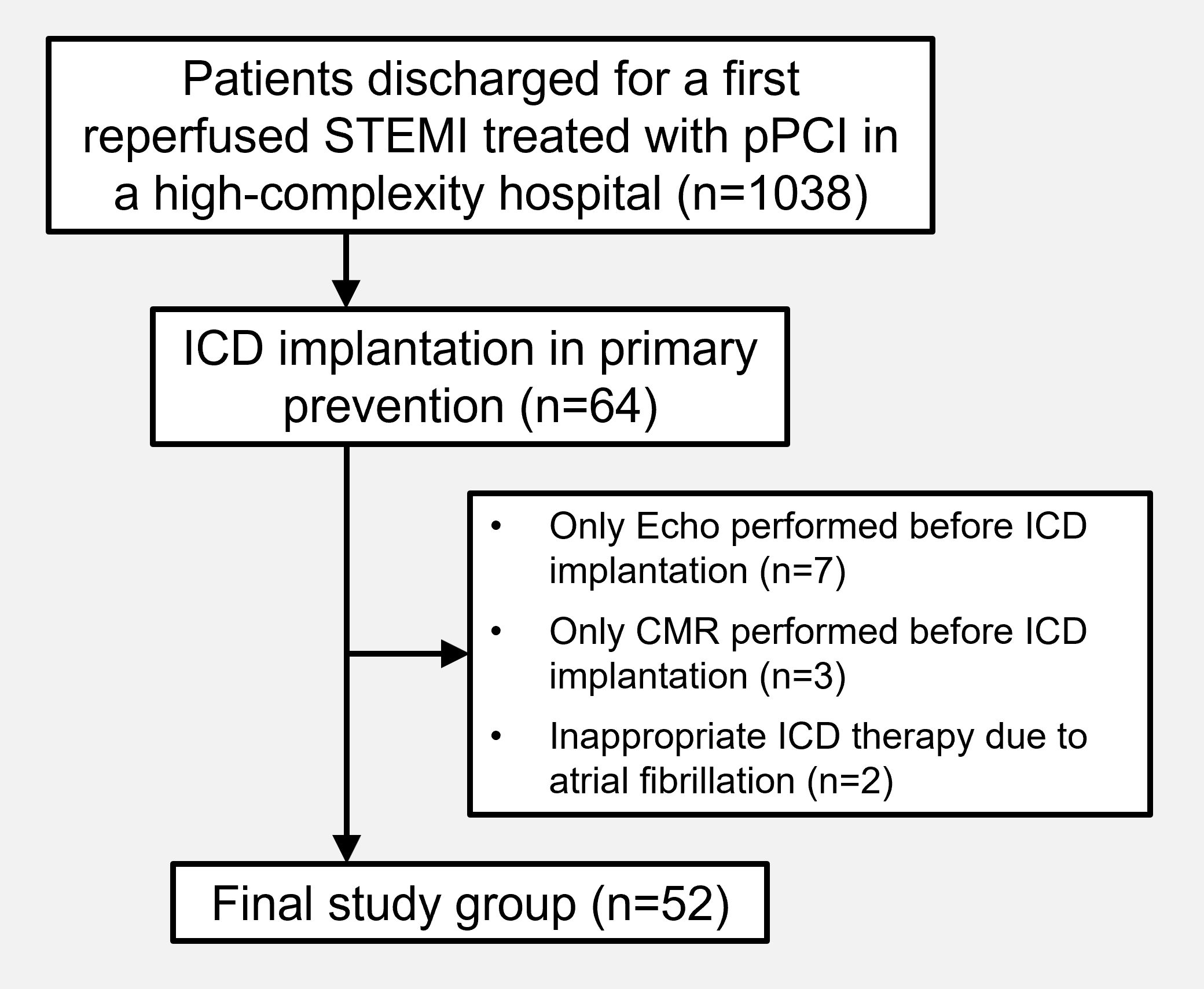


**Supplementary Figure 1.** Flowchart of study patients.

Abbreviations: CMR= Cardiac magnetic resonance. Echo= Echocardiography. ICD= Implantable cardioverter-defibrillator. pPCI= Primary percutaneous coronary intervention.


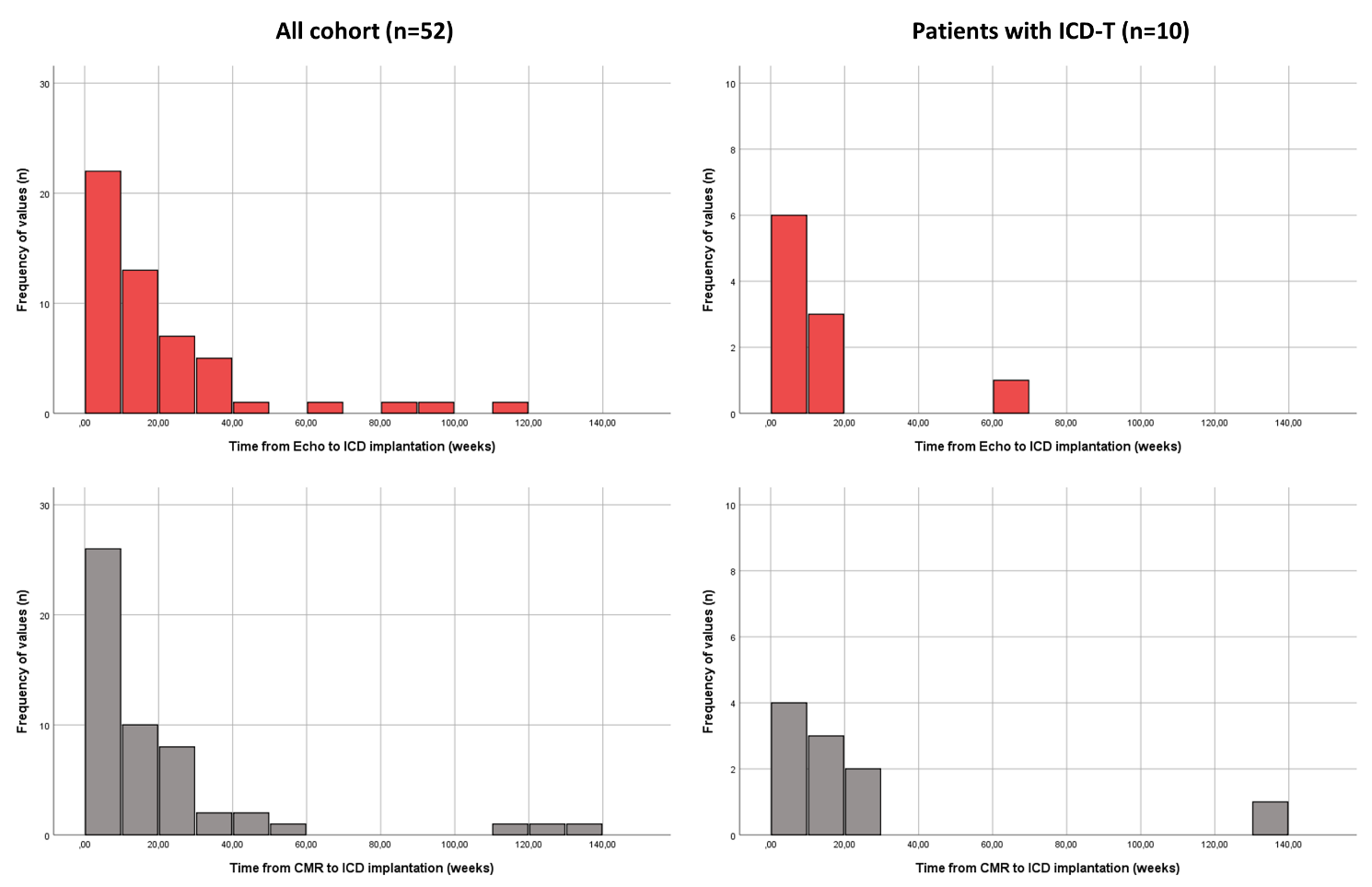


**Supplementary Figure 2.** Histograms of time from Echo and CMR to ICD implantation in the entire cohort and in patients with ICD-T.

Abbreviations: CMR= Cardiac magnetic resonance. Echo= Echocardiography. ICD= Implantable cardioverter-defibrillator. ICD-T= Implantable cardioverter-defibrillator therapies.
